# Association between statin administration and outcome in patients with sepsis: A retrospective study

**DOI:** 10.1515/med-2024-1112

**Published:** 2025-02-04

**Authors:** Jianzhu Zhou, Zeying Feng, Hui Qiu, Tong Li, Xin Huang, Ling Ye, Longjian Huang, Chengjun Guo, Chengxian Guo, Li He

**Affiliations:** Center of Clinical Pharmacology, the Third Xiangya Hospital, Central South University, Changsha, 410013, Hunan, China; Department of Pediatrics, the Third Xiangya Hospital of Central South University, Changsha, 410013, Hunan, China; West Guangxi Key Laboratory for Prevention and Treatment of High-Incidence Diseases, Youjiang Medical University for Nationalities, Baise, 533000, Guangxi, China; School of Applied Mathematics, Guangdong University of Technology, Guangzhou, 510006, Guangdong, China

**Keywords:** sepsis, statin, atorvastatin, mortality, intensive care unit

## Abstract

**Background & aims:**

There was considerable debate regarding the effect of statins administration on the outcome of septic patients. This retrospective study aimed to assess the association between statins administration and mortality in sepsis patients and investigate whether this association differed according to the types of statins.

**Methods:**

We performed a retrospective study based on the electronic ICU Collaborative Research Database, Medical Information Mart for Intensive Care Database, and the Amsterdam University Medical Centers Database. The participants with sepsis were divided as two groups, statins group and non-statins group. The primary endpoint was the all-cause mortality. We utilized logistic regression, propensity score matching (PSM), and sub-analysis to assess the association between statins administration and outcome in patients with sepsis.

**Results:**

A total of 19,327 sepsis patients were enrolled. Among these, 3,721 patients were prescribed statins. Pooled analyses of three databases showed that statin users had a decreased risk of mortality in sepsis as compared with nonusers (OR 0.73, 95% CI 0.66–0.80, *P* < 0.001). Sub-analysis of statin showed that atorvastatin had the most distinct effectiveness in decreasing mortality (OR 0.67, 95% CI 0.59–0.76, *P* = 0.035), whereas pravastatin, simvastatin, and rosuvastatin were not. PSM analysis confirmed these findings for statins (OR 0.75, 95% CI 0.67–0.84, *P* < 0.001) and atorvastatin (OR 0.70, 95% CI 0.59–0.82, *P* < 0.001).

**Conclusions:**

The use of statins could decrease the risk of mortality in patients with sepsis during the hospital period. Among different types of statins, atorvastatin showed the most significant trend to reduce the risk of mortality in patients with sepsis.

## 1 Introduction

Sepsis, a definition of organ dysfunction caused by a dysregulated host response to infection from the guideline of 2021 Surviving Sepsis Campaign [1], is associated with high mortality of intensive patients and hospital case-fatality rate in the ICU can exceed 40% [2]. Furthermore, sepsis is costly to treat, and consumes a lot of medical resource. Consequently, sepsis has rapidly emerged as a significant global health burden. The pathogenesis of sepsis involves the excessive release of pro-inflammatory mediators in infected patients, leading to inflammation and the clinical manifestation of systemic inflammatory response syndrome (SIRS). On this basis, the compensatory anti-inflammatory response (CARS) has failed to act. This imbalance between SIRS and CARS can result in immunoparalysis, disruption of homeostasis, and potentially progress to multiorgan dysfunction [3]. Statins are inhibitors of the hydroxymethylglutaryl-CoA reductase enzyme, have a significant effect of lowering cholesterol, and widely used in hypercholesterolemia and the prevention of cardiovascular disease [4]. Recent studies have revealed potential antibacterial [5], anti-inflammatory [6], and immune modulatory effects of statins [7]. Ongoing debates persist regarding the potential of statins to enhance outcomes in patients with sepsis. Several studies have indicated that statin therapy may play a potentially beneficial role in outcomes of patients with sepsis [8–10], whereas other literature has reported no association between statin prescription and clinical outcomes in sepsis patients [11–13]. A recent review has suggested that the evidence supporting the effectiveness of statins is insufficient, and further research is required to substantiate these findings [14].

The therapeutic efficacy of statin prescribed for sepsis patients appears to vary based on the specific statin type. A prospective randomized controlled trial showed that atorvastatin administration in patients with severe sepsis significantly reduced the 28-day mortality [10]. In contrast, another randomized controlled clinical trial revealed that rosuvastatin therapy did not improve clinical outcomes in critically ill sepsis patients with acute respiratory distress syndrome (ARDS) and potentially contributed to hepatic and renal dysfunction [12]. Furthermore, a secondary analysis of patients with ARDS and sepsis indicated that simvastatin therapy seemed safe and may have reduced mortality in the low cholesterol group, whereas rosuvastatin treatment was associated with a higher risk of mortality [15]. Consistent with these findings, a cohort study suggested that simvastatin and atorvastatin exhibited greater efficacy than rosuvastatin in enhancing 30-day survival rates [16]. In experimental studies, an animal model demonstrated that pravastatin reduced pulmonary microvascular permeability, leading to improved survival in septic mice [17]. Despite numerous reports on the outcomes of sepsis after using specific statins, it remains unclear which type of statin plays a decisive role in the outcomes of patients with sepsis, and currently there is still a lack of large-scale population studies to explore this.

Furthermore, a preponderance of these studies focused on Asian populations. Notably, the association between specific statin types and sepsis outcomes remained unclear. By addressing these research gaps, we can gather more robust clinical evidence on this association and to provide more targeted and effective treatment strategies, ultimately improving patient outcomes. Therefore, the primary aim of this study was to evaluate the association between statin administration and outcomes in patients with sepsis. The secondary aim was to investigate whether various types of statin make different contributions to outcomes of sepsis, with the goal of identifying the specific statin that had the most significant effectiveness. Specifically, we consolidated the clinical records and medication information for sepsis patients from three publicly accessible critical illness databases, and performed analyses using statistical methods, including logistic regression, propensity score matching (PSM), and subgroup analysis.

## 2 Methods

### 2.1 Data source

In this study, we employed three openly accessible databases for data collection and analysis. Compliance with open standards and protocols throughout the data acquisition and utilization ensured transparency and reproducibility of the obtained data. Specifically, the datasets utilized in this study originated from the Amsterdam University Medical Centers Database (AmsterdamUMCdb), the electronic ICU Collaborative Research Database (eICU), and Medical Information Mart for Intensive Care Database (MIMIC-III CareVue). AmsterdamUMCdb represents the first freely accessible intensive care database from within the European Union containing de-identified health data related to tens of thousands of European intensive care unit admissions. It contains extensive clinical data derived from 23,106 admissions involving 20,109 patients, spanning from 2003 to 2016 [18]. The eICU, a large multi-center critical care database, is made available by Philips Healthcare in collaboration with the MIT Laboratory for Computational Physiology. It contains 139,367 unique patients admitted from many intensive care units across the United States during the period of 2014–2015. Furthermore, it preserves data related to more than 200,000 hospitalized patients’ unit encounters [19]. MIMIC-III is a large, freely accessible, single-center critical care database, which integrates anonymized, comprehensive clinical data of over 40,000 patients admitted to the Beth Israel Deaconess Medical Center in Boston, Massachusetts from 2001 to 2012 [20]. The hospitals serving as the sources for the three databases constitute entirely independent datasets collected from a substantial number of hospitals situated in either the United States or the Netherlands.

### 2.2 Study population

#### 2.2.1 Inclusion criteria

Patients with any of the sepsis-related diagnoses were included primarily by searching, identifying, and evaluating clinical data in databases. The diagnostic codes utilized adhere to the standards outlined in the International Classification of Diseases, Ninth Revision (ICD-9). According to the Third International Consensus on the Definition of Sepsis and Septic Shock (Sepsis-3) [21], sepsis is defined as a life-threatening organ dysfunction caused by a dysregulated host response to infection.

#### 2.2.2 Exclusion criteria

Certain special populations, including children, the elderly, pregnant women, AIDS, malignant neoplasms, etc., may contribute a potential interference or risk to the study results due to their specific physiological and pharmacokinetic characteristics. Patients with a hospital stay duration of less than 1 day may struggle to obtain abundant clinical information, thereby compromising the integrity and quality of the data. Patients with multiple hospital admissions may introduce bias in the results due to the presence of duplicated clinical data. Consequently, the exclusion criteria are formulated as follows: (1) age < 18; (2) age over the upper limit of the database, displayed as “>80” (AmsterdamUMCdb) or “>89” (eICU and MIMIC-III), where the specific number cannot be known; (3) pregnant or lactating females; (4) AIDS; (5) malignant neoplasms; (6) ICU stay < 1 day; and (7) non-first admission.

### 2.3 Assessment of statin use

Statins were defined according to the Anatomical Therapeutic Chemical classification system. By reviewing the database, searching for information on drugs from the prescription table to determine whether statins were administered to patients with sepsis. The seven globally prescribed statins are atorvastatin, fluvastatin, lovastatin, pitavastatin, pravastatin, rosuvastatin, and simvastatin, which will be used for the subgroup analysis.

### 2.4 Ascertainment of outcomes

The primary endpoint was all-cause mortality in patients with sepsis, and the cause of death was determined according to the ICD-9. By reviewing databases, the information of in-hospital death could be found in the patient section.

### 2.5 Extraction of covariates

Three databases were reviewed to search, collect, and collate confounding factors that may impact the outcomes of septic patients. On account of the incomplete and inconsistent hospitalization information of septic patients across three databases, we used the mean to replace variables with missing values of less than 20% of the total; however, variables with more than 20% missing values were excluded. Age was an independent predictor of mortality in adult sepsis [22]. Gender, as demographic variable, was associated with risk of death among the ICU for patients with severe sepsis [23]. Appropriate insulin therapy was essential for septic patients with hyperglycemia [24]. Norepinephrine, as the first-line vasopressor, plays a significant role in outcomes of adults with septic shock [1]. Maintenance dialysis was an independent predictor of mortality in patients with severe sepsis [25]. Patients with sepsis who required mechanical ventilation were at higher risk of mortality in ICU [26]. Leukocyte kinetics was a valuable prognostic marker in sepsis, with decreasing white blood cell (WBC) counts correlating with longer survival [27]. The severity of kidney injury, assessed by creatinine levels, was associated with in-hospital mortality in sepsis [28]. Consequently, the following relevant variables were selected for analysis: (1) demographic characteristics: age and gender; (2) biochemical parameters: leukocyte count, serum creatinine, and mean blood glucose levels, recorded at the initial measurement post-admission; (3) concomitant medication use: administration of norepinephrine or insulin; and (4) therapeutic interventions: utilization of mechanical ventilation (intubated) and renal replacement therapy (dialysis) during hospitalization. Variations in collection methods were observed when extracting data from the three databases. First, the AmsterdamUMCdb database was in Dutch, requiring the translation of search terms into Dutch. Second, some pertinent variables were missing from certain databases. For example, the AmsterdamUMCdb lacked records of comorbidities within admission diagnoses. Third, retrieving the same variable may require different templates across databases. For instance, vital signs and biochemical indicators extracted from AmsterdamUMCdb and eICU were accessible within a unified section (“listitem” and “lab”), while in MIMIC-III, these were organized into two distinct sections. Additionally, MIMIC-III required an initial search for “ITEMID,” followed by the input of this “ITEMID” into the corresponding section to extract data, whereas the other two databases simply required the input of keywords. Finally, the units used for the same variable may have differed across databases, necessitating unit conversion.

### 2.6 Statistical analysis

We conducted a normality test (D’Agostino test), followed by a descriptive analysis of the data. Continuous variables were reported as mean (standard deviation), and compared using independent samples *t*-test for each dataset. Categorical variables were presented as frequencies (percentages) and compared using the Chi-square test. Two statistical analysis models were established. The first model, termed the “crude model,” was a simple regression model considering only statin use and mortality. The second model, referred to as the “multivariable adjusted model,” was constructed using covariates common to the three databases and potentially influencing the outcome. Specifically, the independent variables included in this multivariable adjusted model were age, gender, insulin use, norepinephrine administration, dialysis, intubation, WBC count, creatinine levels, and mean blood glucose levels. Multivariable binary logistic regression was conducted to assess the association between in-hospital therapeutic outcomes and statin use among septic patients during hospitalization. For each of the three databases, 95% confidence interval (CI) and odds ratio (OR) were calculated. Pooled the three databases and performed the regression analysis again, obtaining overall 95% CI and OR. Subgroup analyses for different types of statins were conducted using the aforementioned method. PSM was utilized to enhance the robustness of our research findings. In this study, the PSM model utilized a one-to-one nearest neighbor matching algorithm with a caliper width of 0.02. We evaluated matching efficiency by comparing the post-PSM differences (*P*-values) between the two groups using chi-square or *t*-tests. The *P*-values for all baseline characteristics in both groups exceeded 0.05, suggesting that the baseline characteristics were well matched between the groups. In the PSM cohort, we conducted subgroup analyses to not only examine the correlation between statin type and mortality but also to ascertain whether the association between statin administration and in-hospital mortality is modulated by factors including gender, age, norepinephrine administration, insulin usage, dialysis necessity, and tracheal intubation status. Statistical significance was defined as *P*-values <0.05 (two-tailed). Statistical analyses were conducted using IBM SPSS Statistics version 22 and R version 4.4.0.

## 3 Results

### 3.1 Baseline characteristics of included patients with sepsis

**Figure 1:**
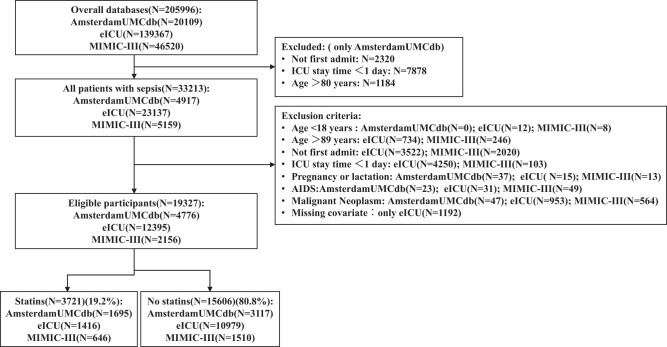
Flowchart of participant selection.

This study encompassed 205,996 critically ill patients from AmsterdamUMCdb (*N* = 20,109), eICU (*N* = 139,367), and MIMIC-III (*N* = 46,520). According to the “Sepsis-3” diagnostic criteria and the inclusion criteria of this study, a total of 19,327 patients with sepsis were deemed eligible for analysis, distributed across AmsterdamUMCdb (*N* = 4,776), eICU (*N* = 12,395), and MIMIC-III (*N* = 2,156). Among these, 3,721 statin users were identified in AmsterdamUMCdb (*N* = 1,659), eICU (*N* = 1,416), and MIMIC-III (*N* = 646) (Figure 1 for the study flowchart). The baseline characteristics of patients with sepsis are presented in Table 1. Compared to non-statin users (*N* = 15,606), statin users across all three databases (*N* = 3,721) exhibited significant differences in older age, greater proportion of insulin users, and higher average blood glucose levels. The pooled analysis results revealed significant differences between the two groups in all characteristics except for dialysis, with statin users having a lower proportion of males, a higher proportion of insulin and norepinephrine users, a greater proportion of patients undergoing mechanical ventilation, lower levels of WBCs and blood creatinine, and higher mean blood glucose levels.

**Table 1:** Baseline characteristics by the use of statins in patients with sepsis

Characteristics	AmsterdamUMC (*N* = 4,776)		*P*-value	eICU (*N* = 12,395)		*P*-value	MIMICIII (*N* = 2,156)		*P*-value	Pool (*N* = 19,327)		*P*-value
	Non-statins group (*N* = 3,117)	Statins group (*N* = 1,659)		Non-statins group (*N* = 10,979)	Statins group (*N* = 1,416)		Non-statins group (*N* = 1,510)	Statins group (*N* = 646)		Non-statins group (*N* = 15,606)	Statins group (*N* = 3,721)	
Mean (SD) age, years	56.2 (16.1)	66.1 (9.5)	**<0.001**	63.9 (15.8)	69.4 (12.1)	**<0.001**	61.5 (17.0)	71.1 (11.8)	**<0.001**	62.2 (16.2)	68.2 (11.1)	**<0.001**
**Gender**			**<0.001**			0.129			0.523			**<0.001**
Male, %	62.8	73.5		49.2	47.1		55.1	56.6		48.4	41.7	
Any use of insulin, %	56.2	75.1	**<0.001**	31.9	65.6	**<0.001**	69.4	80.1	**<0.001**	40.4	72.4	**<0.001**
Any use of norepinephrine, %	52.1	51.6	0.754	21.1	23.3	0.059	44.4	42.5	0.423	29.5	39.2	**<0.001**
Dialysis, %	1.1	0.6	0.076	5.5	7.2	**0.007**	11.7	13.9	0.167	5.2	5.4	0.589
Intubated, %	70.4	85.1	**<0.001**	18.8	15.6	**0.003**	28	29.4	0.510	30	49	**<0.001**
Mean (SD) WBC, 10^9^/L	11.8 (9.2)	10.9 (5.1)	**<0.001**	15.7 (10.1)	15.7 (9.0)	0.852	13.9 (8.7)	14.0 (7.6)	0.958	14.7 (9.9)	13.3 (7.6)	**<0.001**
Mean (SD) creatinine, mg/dL	1.2 (1.5)	1.1 (0.9)	0.281	2.0 (1.8)	2.2 (2.0)	**<0.001**	1.8 (1.5)	2.1 (1.8)	**<0.001**	1.8 (1.7)	1.7 (1.6)	**0.030**
Mean (SD) mean blood glucose, mg/dL	142.1 (27.4)	154.2 (26.8)	**<0.001**	140.8 (50.1)	152.3 (56.4)	**<0.001**	129.9 (37.0)	139.2 (34.3)	**<0.001**	140.0 (45.4)	150.9 (42.0)	**<0.001**

Data are presented as mean ± standard deviation or count (percentage). WBC: white blood cell. *P*-values less than 0.05 are in bold.

### 3.2 Association between statins and mortality in patients with sepsis

The univariate analysis of the AmsterdamUMC database indicated that, compared to the non-statin group, the statin group had a reduced risk of in-hospital mortality (OR 0.88, 95% CI 0.77–1.02), although this difference was not statistically significant. After adjusting for covariates, a significant association between statin use and mortality in patients with sepsis was observed. The analysis revealed that patients with sepsis who received statins, had improved outcomes, with reduced in-hospital mortality (OR 0.71, 95% CI 0.60–0.83, *P* < 0.001).

The univariate analysis found that, compared to the other two databases, the statin group in eICU demonstrated the greatest risk reduction in hospital mortality (OR 0.69, 95% CI 0.58–0.82), and the difference was statistically significant (*P* < 0.001). The multivariable analysis confirmed a similar trend. This association was intensified somewhat, and remained significant after multivariable adjustment as well (OR 0.61, 95% CI 0.50–0.73, *P* < 0.001).

The univariate analysis of MIMIC-III revealed that the mortality risk in the statin group was comparable to that in the AmsterdamUMCdb (OR 0.87, 95% CI 0.71–1.07), with no statistically significant difference. The multivariate adjusted analysis demonstrated a statistically significant reduced mortality risk in the statin group (OR 0.62, 95% CI 0.49–0.78, *P* < 0.001).

When the three databases were pooled for analysis, the univariate analysis unexpectedly revealed that the reduced mortality risk trend in the statin group observed in the individual databases was no longer present, with a non-significant trend toward increased mortality risk (OR 1.06, 95% CI 0.97–1.16). Notably, the multivariate adjusted analysis confirmed a significant trend toward reduced mortality risk in the statin group (OR 0.73, 95% CI 0.66–0.80, *P* < 0.001) (Table 2).

**Table 2:** Association between statin and mortality in sepsis patients

		Crude model		Multivariable adjusted model	
	Cases/participants	OR (95% CI)	*P*-value	OR (95% CI)	*P*-value
**AmsterdamUMCdb**					
Non-statins	809/3,117	1.00 (Reference)	Reference	1.00 (Reference)	Reference
Statins	394/1,659	0.88 (0.77–1.02)	0.095	0.71 (0.60–0.83)	**<0.001**
**eICU**					
Non-statins	1,616/10,979	1.00 (Reference)	Reference	1.00 (Reference)	Reference
Statins	151/1,416	0.69 (0.58–0.82)	**<0.001**	0.61 (0.50–0.73)	**<0.001**
**MIMIC-III**					
Non-statins	465/1,510	1.00 (Reference)	Reference	1.00 (Reference)	Reference
Statins	181/646	0.87 (0.71–1.07)	0.198	0.62 (0.49–0.78)	**<0.001**
**Pool**					
Non-statins	2,890/15,606	1.00 (Reference)	Reference	1.00 (Reference)	Reference
Statins	726/3,721	1.06 (0.97–1.16)	0.163	0.73 (0.66–0.80)	**<0.001**

Multivariable adjusted model included age, gender, insulin, norepinephrine, dialysis, intubation, WBC (white blood cell) count, creatinine levels, and mean blood glucose levels. OR: odds ratio; CI: confidence interval. *P*-values less than 0.05 are in bold.

### 3.3 Subgroup analysis of statins used in patients with sepsis

The univariate analysis of the AmsterdamUMC database indicated that, compared to the non-statin group, patients who used atorvastatin or rosuvastatin exhibited a reduced risk of mortality, whereas those prescribed pravastatin or simvastatin showed a potential increase in mortality. However, these differences did not reach statistical significance. After adjusting for covariates, significant associations were observed between various statin types and mortality in patients with sepsis. A trend toward decreased mortality risk was observed for all statin types, including atorvastatin, rosuvastatin, pravastatin, and simvastatin. Notably, statistically significant differences were observed in the atorvastatin group (OR 0.73, 95% CI 0.59–0.91, *P* = 0.005) and the rosuvastatin group (OR 0.51, 95% CI 0.26–0.98, *P* = 0.045).

The univariate analysis in eICU revealed that, compared to the non-statin group, the atorvastatin, pravastatin, and simvastatin groups all exhibited trends toward reduced mortality risk in sepsis patients, with the atorvastatin group showing statistical significance (OR 0.68, 95% CI 0.56–0.82, *P* < 0.001). The multivariate adjusted analysis confirmed a slight intensification of the trend toward decreased mortality among various statins, with the atorvastatin group maintaining statistical significance (OR 0.61, 95% CI 0.50–0.75, *P* < 0.001).

The univariate analysis of MIMIC-III revealed that the pravastatin and simvastatin groups exhibited a reduced risk of mortality, while the atorvastatin and rosuvastatin groups showed an increased risk. However, only the pravastatin group demonstrated statistical significance (OR 0.33, 95% CI 0.14–0.79, *P* = 0.013). The lovastatin group had too few cases (*N* = 3) for meaningful analysis. The multivariate adjusted analysis demonstrated a decreased risk of mortality for the atorvastatin (OR 0.74, 95% CI 0.57–0.98, *P* = 0.035), pravastatin (OR 0.24, 95% CI 0.10–0.61, *P* = 0.003), and simvastatin (OR 0.70, 95% CI 0.50–0.97, *P* = 0.035) groups, with all three groups showing statistical significance. Despite a reduced trend in increasing mortality risk in the rosuvastatin group, this trend was not statistically significant.

We pooled and analyzed data from the three databases. The univariate analysis demonstrated that the atorvastatin group reduced the mortality risk and the difference was statistically significant (OR 0.88, 95% CI 0.78–0.99, *P* = 0.043). Conversely, the simvastatin group demonstrated a statistically significant trend toward increased mortality risk (OR 1.40, 95% CI 1.23–1.60, *P* < 0.001). However, no statistically significant increase in mortality risk was observed for the pravastatin and rosuvastatin groups. After adjusting for covariates, the analysis revealed that different types of statins reduced the risk of mortality, albeit only the atorvastatin group reached statistical significance (OR 0.67, 95% CI 0.59–0.76, *P* = 0.035). Notably, after multivariable adjustment, simvastatin shifted from an elevated to reduced mortality risk, albeit this change was not statistically significant (Table 3).

**Table 3:** Subgroup analysis of statin types in sepsis patients

		Crude model		Multivariable adjusted model	
	Cases/participants	OR (95% CI)	*P*-value	OR (95% CI)	*P*-value
**AmsterdamUMCdb**					
Statins	394/1,659				
Atorvastatin	136/618	0.81 (0.66–1.00)	0.051	0.73 (0.59–0.91)	**0.005**
Pravastatin	31/111	1.15 (0.75–1.75)	0.501	0.99 (0.63–1.56)	0.997
Simvastatin	226/879	1.03 (0.87–1.22)	0.693	0.85 (0.71–1.02)	0.093
Rosuvastatin	12/74	0.57 (0.30–1.06)	0.077	0.51 (0.26–0.98)	**0.045**
**eICU**					
Statins	151/1,416				
Atorvastatin	123/1,175	0.68 (0.56–0.82)	**<0.001**	0.61 (0.50–0.75)	**<0.001**
Pravastatin	4/58	0.44 (0.16–1.22)	0.118	0.35 (0.12–1.00)	0.051
Simvastatin	26/201	0.89 (0.58–1.35)	0.590	0.78 (0.51–1.20)	0.264
**MIMIC-III**					
Statins	181/646				
Atorvastatin	109/358	1.02 (0.80–1.31)	0.827	0.74 (0.57–0.98)	**0.035**
Pravastatin	6/47	0.33 (0.14–0.79)	**0.013**	0.24 (0.10–0.61)	**0.003**
Simvastatin	66/247	0.83 (0.62–1.12)	0.238	0.70 (0.50–0.97)	**0.035**
Rosuvastatin	7/18	1.49 (0.57–3.86)	0.410	1.23 (0.45–3.38)	0.677
Lovastatin	0/3	NA	NA	NA	NA
**Pool**					
Statins	726/3,721				
Atorvastatin	368/2,151	0.88 (0.78–0.99)	**0.043**	0.67 (0.59–0.76)	**0.035**
Pravastatin	41/216	1.01 (0.72–1.43)	0.918	0.70 (0.49–1.00)	0.053
Simvastatin	318/1,327	1.40 (1.23–1.60)	**<0.001**	0.96 (0.84–1.11)	0.632
Rosuvastatin	19/92	1.13 (0.68–1.87)	0.632	0.76 (0.45–1.29)	0.323
Lovastatin	0/3	NA	NA	NA	NA

Multivariable adjusted model included age, gender, insulin, norepinephrine, dialysis, intubation, WBC (white blood cell) count, creatinine levels, and mean blood glucose levels. OR: odds ratio; CI: confidence interval. NA: not applicable. *P*-values less than 0.05 are in bold.

### 3.4 PSM and subgroup analysis

**Figure 2:**
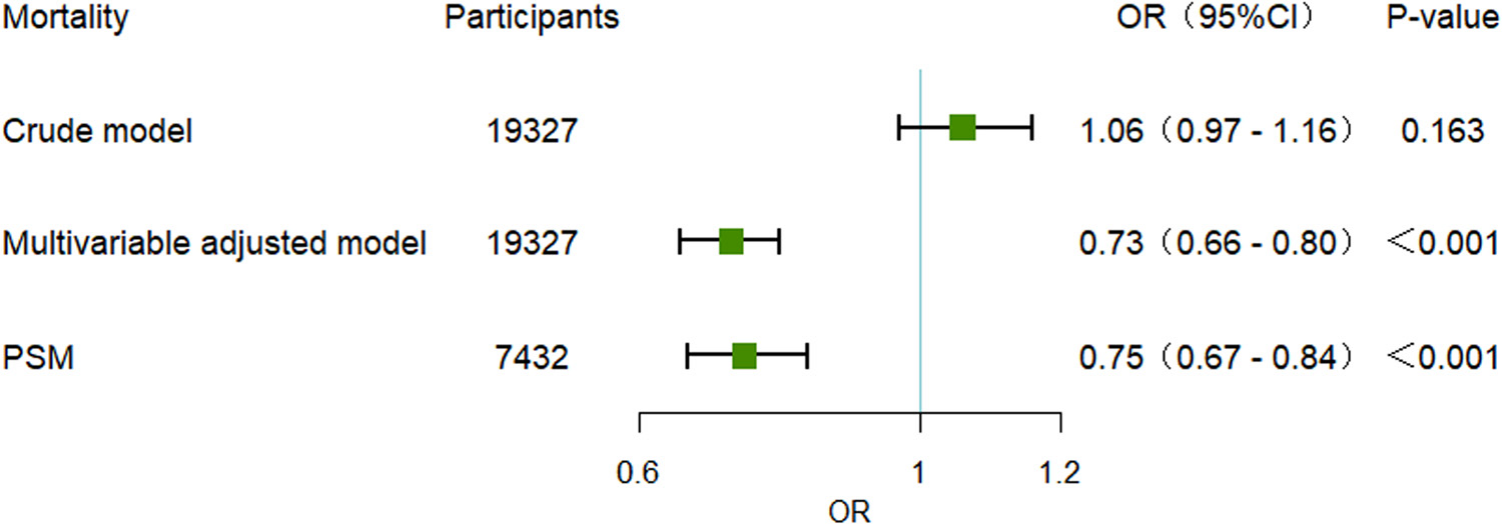
Association between statin use and mortality of sepsis patients before and after PSM. OR: odds ratio; CI: confidence interval; crude model: without adjustment; multivariable adjusted model: adjusted for age, gender, insulin, norepinephrine, dialysis, intubation, WBC (white blood cell) count, creatinine levels, and mean blood glucose levels. PSM: propensity score matching.

**Figure 3:**
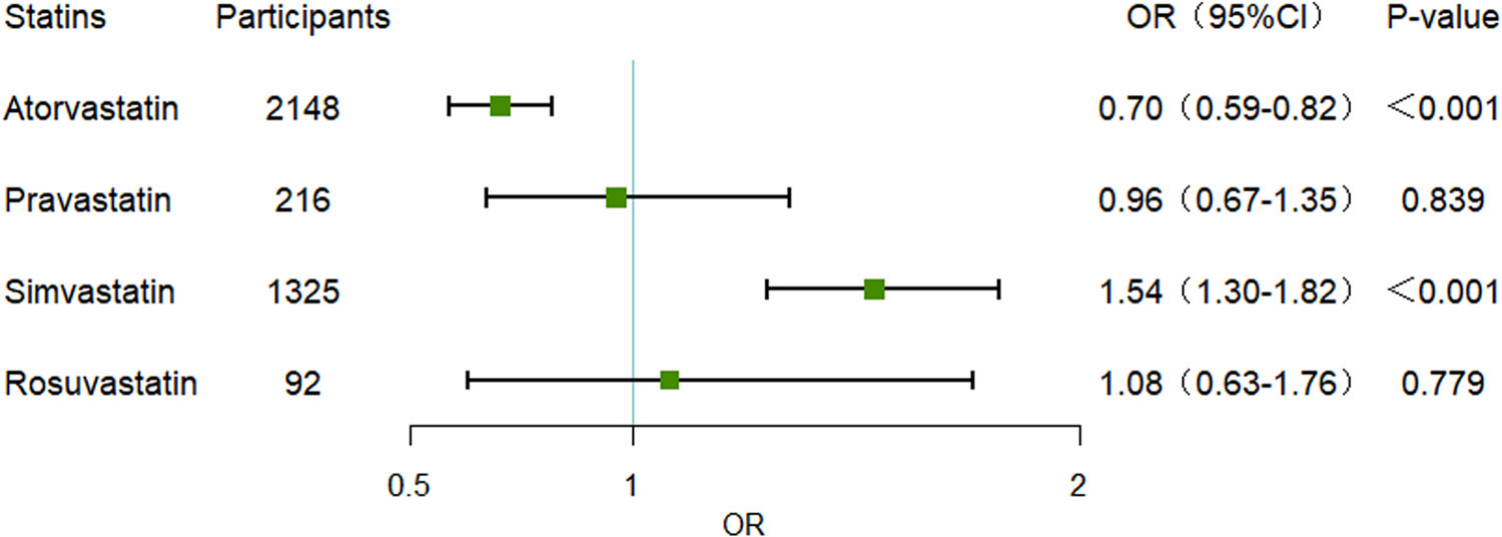
Subgroup analysis of statin types in sepsis patients after PSM. OR: odds ratio; CI: confidence interval; PSM: propensity score matching.

**Figure 4:**
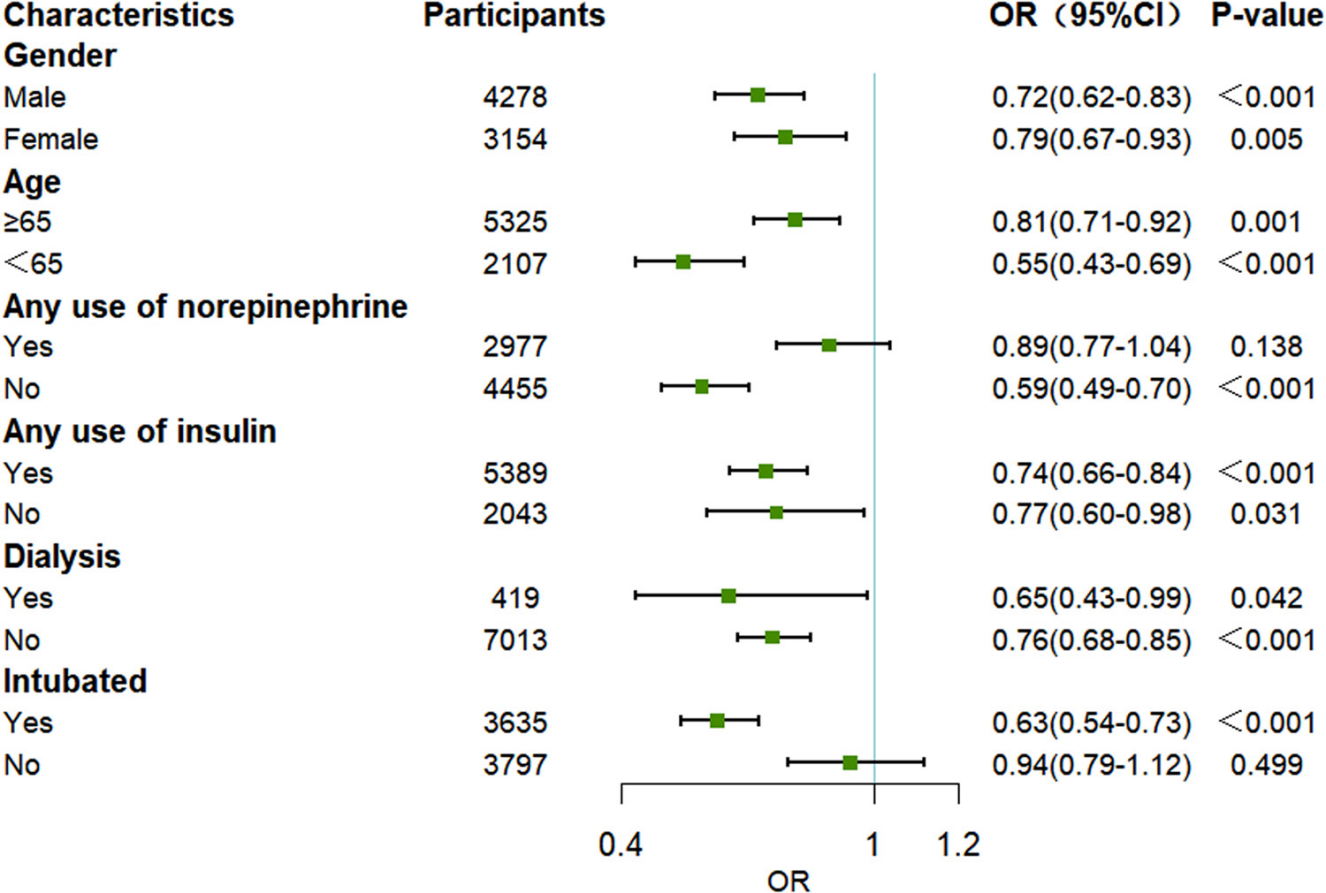
Association between statin use and mortality of sepsis patients in subgroup after PSM. OR: odds ratio; CI: confidence interval; PSM: propensity score matching.

After PSM, 3,716 statin users and 3,716 non-statin users were included in the final analysis. The baseline characteristics were well balanced between the two groups, with all *P*-values exceeding 0.05 (Table 4). Univariate logistic regression within the PSM cohort demonstrated a significant reduction in mortality among sepsis patients associated with statin use (OR 0.75, 95% CI 0.67–0.84, *P* < 0.001) (Figure 2). In the PSM cohort, different types of statins exerted varying effects on patient mortality. Atorvastatin and pravastatin were associated with a reduced risk of mortality, whereas simvastatin and rosuvastatin were associated with an increased risk of mortality. However, only atorvastatin (OR 0.70, 95% CI 0.59–0.82, *P* < 0.001) and simvastatin (OR 1.54, 95% CI 1.30–1.82, *P* < 0.001) demonstrated statistically significant differences (Figure 3). Furthermore, subgroup analyses were conducted on the PSM cohort. Across different subgroups, a consistent trend was observed: statin use was associated with a reduced in-hospital mortality rate among sepsis patients. Moreover, this association was statistically significant (*P* < 0.05) in all subgroups except for those receiving norepinephrine and without intubation (Figure 4).

**Table 4:** Baseline characteristics of sepsis patients after PSM

Post-matched characteristics	Non-statins *N* = 3,716	Statins *N* = 3,716	*P*-value
Mean (SD) age, years	68.4 (12.8)	68.2 (11.2)	0.596
**Gender**			0.260
Male, %	56.9	58.2	
Any use of insulin, %	72.6	72.3	0.815
Any use of norepinephrine, %	40.7	39.3	0.193
Dialysis, %	5.8	5.4	0.513
Intubated, %	48.8	48.9	0.945
Mean (SD) WBC, 10^9^/L	13.4 (8.4)	13.3 (7.6)	0.506
Mean (SD) creatinine, mg/dL	1.8 (1.6)	1.8 (1.7)	0.638
Mean (SD) mean blood glucose, mg/dL	150.3 (49.0)	150.9 (42.1)	0.603

Data are presented as mean ± standard deviation or count (percentage). WBC: white blood cell.

## 4 Discussion

A pooled analysis of three population-based databases revealed that statin treatment during hospitalization was associated with a 27% reduction in the risk of mortality in patients with sepsis compared to those who did not receive statin therapy. Further analysis of different statin types showed that atorvastatin treatment was associated with a 33% reduction in mortality risk. After conducting PSM analysis, the beneficial effect of statins on improving outcomes remained evident. Additionally, subgroup analyses confirmed that this trend of reduced mortality remained consistent across various subgroups.

Although statins have been widely investigated, their therapeutic efficacy in sepsis remains a contentious issue. Pertzov et al. [29] and Deshpande et al. [30], each published a meta-analysis, respectively, pointing out that statins did not reduce 30-day all-cause mortality in a subgroup of patients with severe sepsis. Goodin et al. [31] also reported no beneficial effect of statin use on hospital mortality among patients admitted with sepsis. However, these studies had limited sample sizes, and the meta-analysis encompassed patients who developed sepsis during their hospital stay. It might encompass non-sepsis patients, potentially biasing the true effect of statins on patients. Therefore, these limitations may have underestimated the true impact of statins on sepsis patients. Conversely, an increasing number of studies supported the notion that statins may improve survival in patients with sepsis. Ghayda et al. [32] identified a protective effect of statins on bacteremia/sepsis-related mortality, albeit with a weak level of evidence. Lee et al. [33] revealed that preadmission statin therapy was associated with a 12% reduction in mortality in a cohort study of sepsis development. The results of this retrospective study were consistent with these findings, demonstrating a significant trend toward reduced hospital mortality risk in patients with sepsis following multivariate adjustment. Ou et al. [34] utilized PSM to analyze a population-based cohort study, where high-potency statins, defined as rosuvastatin ≥10 mg, atorvastatin ≥20 mg, and simvastatin ≥40 mg, were more effective in improving sepsis outcomes than low-potency statins (all other dose of statin treatments). Lee et al. [16] investigated the drug-specific effects of statins and their correlation with lipid-lowering potency. Their results suggested that, compared with non-users, atorvastatin and simvastatin were associated with improved 30-day survival, whereas rosuvastatin was not. Ouellette et al. [35] demonstrated that patients who had received atorvastatin prior to hospitalization had a significantly lower mortality rate than those who received simvastatin. Furthermore, atorvastatin treatment prior to sepsis was associated with improved in-hospital outcomes in this study. Liang et al. [36] also reported that atorvastatin was associated with better 30-day outcomes than simvastatin in patients with sepsis. These findings seemed to indicate that the effect of statins on patients with sepsis may vary among different statin types. Most studies with positive findings were based on large-scale cohort studies, many of which specifically focused on Asian populations. Yang et al. [37] suggested that to achieve statistical significance in studies examining sepsis mortality, a sample size exceeding 6,000–7,000 patients was necessary. In this study, the sepsis sample size from Europe and the United States exceeded 19,000 patients, which may allow the results to more accurately reflect the true impact of statins in these regions. Our subgroup analysis results, pooled from the data of this study, were consistent with previous reports, indicating that atorvastatin, a highly potent statin, significantly reduced the risk of mortality in sepsis patients. In our PSM cohort, it is also apparent that atorvastatin significantly improves in-hospital outcomes compared to other statin types. We observed that not only rosuvastatin in AmsterdamUMCdb, but also pravastatin and simvastatin in MIMIC-III demonstrated a statistical significance in reducing risk of mortality. However, due to the limited sample size, the results were not very reliable. In the pooled analysis, these statin types did not exhibit a statistically significant effect in improving patient outcomes.

The reduction in sepsis-related mortality associated with statin use during hospitalization was likely attributable to their pleiotropic effects. The mechanisms underlying these pleiotropic effects of statins encompassed enhancements in endothelial function and vascular tone, plaque stabilization, anti-inflammation actions, anti-thrombosis properties, and reduction of oxidative stress [38]. On the one hand, statins executed anti-inflammatory effects by modulating upstream intracellular signaling pathways [39], on the other hand, cytokines such as TNF, IL-1β, and IL-6, were pivotal in the manifestation of SIRS and might serve as prognostic biomarkers in sepsis [40]. Atorvastatin significantly decreased the levels of IL-1β, TNF-α, and IL-6 [41], while simvastatin reduced the expression of IL-6 and IL-8 in peripheral blood mononuclear cells [42]. Statins also possessed potential anti-infective properties [4] by influencing the biosynthesis of cholesterol, isoprenoid, and lipid compounds biosynthesis, which were crucial for the cellular signaling and structure of pathogens. However, the precise mechanisms underlying the differences in antimicrobial activity among different statin types remained elusive, and the chemical structures and properties of the various statins may influence their antibacterial targeting. Existing literature suggested that the antimicrobial activity of statins was associated with specific chemical functional groups, for instance, the presence of structures such as hydrophobic moieties, lactone ring, or dihydroxy acid within the ring system was closely correlated with anti-*Staphylococcus aureus* activity [43]. The lipophilic nature of simvastatin or atorvastatin may facilitate better binding to bacterial cell walls compared to the hydrophilic rosuvastatin. Gram-negative bacteria were predominant among septic pathogenic pathogens, followed by Gram-positive bacteria [44]. The most common pathogenic bacteria in bloodstream infections included *Escherichia coli*, *Klebsiella pneumoniae*, *S. aureus*, and *Streptococcus pyogenes* [45]. Atorvastatin and simvastatin demonstrated greater antibacterial effects against Gram-positive bacteria, including methicillin-sensitive *S. aureus* and methicillin-resistant *S. aureus*. Additionally, atorvastatin exhibited higher antibacterial activity against Gram-negative bacteria, such as *E. coli*, *Proteus mirabilis*, and *Enterobacter cloacae* [46]. Sepsis pathophysiology indicated that the homeostatic imbalance between SIRS and CARS contributed to the clinical progression of multiple organ dysfunction. The pleiotropic effects of statins, including antimicrobial, anti-inflammatory, and immunomodulatory properties, might underlie their potential mechanism of action in sepsis. Atorvastatin, characterized by high efficiency, lipophilicity, and a broad antimicrobial spectrum, might distinguish itself from other statin types and exhibit a trend toward significantly reducing mortality risk in sepsis patients.

This study reported on the association between statin use and in-hospital mortality risk among sepsis patients, drawing data from three intensive care databases. We observed consistent associations across three independent critical illness databases from the United States and the Netherlands, indicating that statins improve outcomes for sepsis patients. Specifically, atorvastatin demonstrated a significant trend toward reducing the risk of in-hospital mortality. The findings of this study provided a potential basis for considering statins in the treatment of sepsis patients. When the indications for statin therapy are met, clinicians may consider prescribing statins to sepsis patients, as this may potentially improve patient prognosis. When selecting a statin, atorvastatin should be prioritized due to its significant mortality-reducing effect, which is likely to exert greater beneficial effects in sepsis patients. However, caution should be exercised when choosing simvastatin.

There were several limitations in this study. First, the retrospective design limited causality inference. Additionally, the study excluded children, pregnant women, and other special populations, necessitating further research to validate statin use and its impact on sepsis prognosis in these groups. Second, subjectivity might have influenced data collection, and earlier datasets might not account for the impact of COVID-19. Moreover, the absence of data from Asia, Africa, or other regions, limited the broad representativeness and generalizability of the results. Inconsistencies among the three databases could result in omitted information regarding certain sepsis patients. Third, the retrospective nature of the study may have introduced confounding factors, such as the comorbidities and selection bias which may affect the results. Furthermore, potential confounders, including microbial sources, inflammatory cytokine levels, genetic variations affecting statin efficacy, and infection sites, were not accounted for in the analysis, introducing uncertainties. Lastly, statin types varied across databases, and we observed that some statin types had small sample sizes, potentially leading to sampling errors. Based on current evidence, further randomized controlled trials, prospective studies are warranted to confirm whether the use of statins in sepsis can benefit patients through pleiotropic effects during hospitalization.

## 5 Conclusion

The use of statins had the potential to decrease the risk of mortality in patients with sepsis during hospitalization. Among the various types of statins, atorvastatin demonstrated the most prominent trend in reducing the risk of mortality among patients with sepsis.
